# When Data Sharing Gets Close to 100%: What Human Paleogenetics Can Teach the Open Science Movement

**DOI:** 10.1371/journal.pone.0121409

**Published:** 2015-03-23

**Authors:** Paolo Anagnostou, Marco Capocasa, Nicola Milia, Emanuele Sanna, Cinzia Battaggia, Daniela Luzi, Giovanni Destro Bisol

**Affiliations:** 1 Dipartimento di Biologia Ambientale, “Sapienza” Università di Roma, Rome, Italy; 2 Istituto Italiano di Antropologia, Rome, Italy; 3 Dipartimento Biologia e Biotecnologie “Charles Darwin”, “Sapienza” Università di Roma, Rome, Italy; 4 Dipartimento di Scienze della Vita e dell'Ambiente, Università di Cagliari, Cagliari, Italy; 5 Istituto di Ricerche sulla Popolazione e le Politiche Sociali, Consiglio Nazionale delle Ricerche, Rome, Italy; University of Wisconsin, UNITED STATES

## Abstract

This study analyzes data sharing regarding mitochondrial, Y chromosomal and autosomal polymorphisms in a total of 162 papers on ancient human DNA published between 1988 and 2013. The estimated sharing rate was not far from totality (97.6% ± 2.1%) and substantially higher than observed in other fields of genetic research (evolutionary, medical and forensic genetics). Both a *questionnaire*-based survey and the examination of Journals’ editorial policies suggest that this high sharing rate cannot be simply explained by the need to comply with stakeholders requests. Most data were made available through body text, but the use of primary databases increased in coincidence with the introduction of complete mitochondrial and next-generation sequencing methods. Our study highlights three important aspects. First, our results imply that researchers’ awareness of the importance of openness and transparency for scientific progress may complement stakeholders’ policies in achieving very high sharing rates. Second, widespread data sharing does not necessarily coincide with a prevalent use of practices which maximize data findability, accessibility, useability and preservation. A detailed look at the different ways in which data are released can be very useful to detect failures to adopt the best sharing modalities and understand how to correct them. Third and finally, the case of human paleogenetics tells us that a widespread awareness of the importance of Open Science may be important to build reliable scientific practices even in the presence of complex experimental challenges.

## Introduction

Making research data openly accessible to the scientific community is one of the main priorities for the global research system. In fact, there is wide consensus that data sharing may help scientific progress allowing a better exploitation of data and an optimized use of resources in a climate of scientific openness and transparency [1–3]. However, there are also considerable barriers to be overcome, such as the inherent time and economic costs, possible data misuse, ethical issues and conflicts of interest with patenting discoveries [4–6]. Given this tension, the diffusion of robust and effective open data practices should be viewed as an ongoing process which needs to be sustained by a cooperative effort of researchers, governments and other stakeholders [2, 7–11]. Strategies pursued by most academic institutions and funding bodies are mainly based on the development of digital infrastructures [12, 13] and policies [7, 14, 15], while a number of scientific journals has adopted guidelines for data archiving, preservation and sharing [16, 17]. All these top-down initiatives are certainly indispensable. However, they may be empowered by bottom-up approaches such as empirical studies of data sharing practices based on *questionnaire-*based surveys or analyses of data retrievability from scientific literature [14, 18, 19]. Such initiatives may support the Open Science movement by providing quantitative answers to questions which regard norms (are they effective?), motivations (why do researchers choose to share or withhold?) and ways to share data (do they maximize data findability, accessibility, useability and preservation?). Another significant outcome of this kind of study could be the identification of “flagship research fields”, scientific areas of inquiry in which data sharing has already become common practice [20]. Apart from their symbolic value, identifying such positive examples may have a double outcome: (i) identify conditions and practices which may help spread data sharing; (ii) help understand whether and how data openness may contribute to the development of specific research fields. Unfortunately, studies carried out to date have failed to identify such positive examples. However, in the field of genomics, in particular, there are important initiatives in which data sharing has become the norm, such as the Human Genome and Hap-Map projects or the database of Genotypes and Phenotypes [21, 22]. Nevertheless, all empirical studies conducted so far clearly show that when we move the focus from specific projects to the wider scale of research fields, data sharing turns out to be far from being common practice [14, 23–34].

In this study, we analyze data sharing in publications regarding ancient human DNA studies (hereafter referred to as human paleogenetics), a research field of particular interest for empirical investigations due to its high standards in terms of reliability and experimental reproducibility. Differently from most previous studies, we do not simply provide estimates of sharing rates but also consider the spectrum of data sharing modalities, i.e. the different ways (with body text and online primary databases at the two extremes, see “Materials and Methods”) through which data are publicly released. We also combine the analysis of published papers with a *questionnaire*-based survey, showing that that data sharing is common practice in human paleogenetics and that the authors’ awareness of the importance of openness and transparency for scientific progress might have contributed to such behaviour. Thereafter, we compare the results obtained with findings of a previous study conducted in three genetic research fields (evolutionary, forensic and medical genetics) taking into consideration not only data availability but also the modalities in which data are shared. Finally, we argue that the human paleogenetics case study might contribute to the Open Science movement focusing on three points: (i) the possible role of epistemological motivations to achieve high sharing rates; (ii) the usefulness of looking carefully at the modalities in which data are made available to make data sharing robust and effective; (iii) the importance of openness and transparency to build rigorous and reliable scientific practices in the presence of complex experimental challenges.

## Methods

### Basic Definitions

Given their complex nature, it seems opportune to start the description of our protocol of analysis by giving an explicit definition of the meaning of the terms “data” and “sharing” that we adopted throughout the study.

In this research, we focused on different types of polymorphisms (S1 Dataset) relative to mitochondrial DNA, Y chromosome and autosomes plus X chromosome. It should be noted that “data” considered here may be considered derivative of experimental data [35]. In fact, they derive from the manual or electronic processing of raw data obtained using combinations of biochemical methods (e.g. DNA purification, Polymerase Chain Reaction, electrophoresis or Next Generation Sequencing).

Any given dataset was counted as shared if released with a minimum of accompanying information (absolute frequency of each variable and geographic location or dating of the individual/s sampled), and in a format that permits their reuse both in individual (e.g. haplotype or sequence matching) and population analyses (e.g. calculation of intra and inter population differentiation measures) (see below for further details). To resolve the shared/withheld dichotomy we: (i) searched for the data both in papers and in their supplementary material; (ii) when an accession number was given, we checked for the actual data availability; (iii) when no accession number was given in the paper (even when the data was already provided as body text or supplementary material), datasets were anyhow searched for in GenBank using the paper titles as a keyword. Unfortunately, it was not possible to carry out any systematic analysis of the context in which data were created [6], and hence appreciate purpose, reproducibility and quality of experimental results, due to lack of information in the vast majority of papers under examination.

### Data collection and analysis

Our study is based on the scrutiny of papers published between October 1988 and December 2013, which were retrieved from the PubMed database (http://www.ncbi.nlm.nih.gov/pubmed) using 15 combinations of relevant key words (S1 Dataset). The following species were considered: *Homo sapiens*, *Homo neanderthalensis* and *Homo denisovensis*. After removing irrelevant studies (e.g. studies not pertinent to human populations, reviews or meta-analyses), we selected 162 papers containing a total of 207 datasets which were analyzed using an already developed protocol [36].

Further information regarding the experimental procedures (tissues collected, number of laboratories involved, independent replicates of raw data performed) is also provided in S1 Dataset.

Each paper went through two independent procedures of data collection, each performed by an experienced researcher. When conclusions were discordant, consensus was reached with the help of a third researcher who had independently analyzed the papers.

Specific criteria to assign a dataset to the “shared” category were as follows:

- for unilinearly transmitted polymorphisms (mtDNA and Y chromosome): when full haplotypic information of all individual DNAs genotyped and/or sequenced was available; this means that, when more than one type of polymorphism was analyzed (e.g. Single Nucleotide polymorphisms, SNPs, and microsatellites) it had to be possible to reconstruct compound haplotypes.
- for autosomal polymorphisms: when the genetic profile for all loci genotyped/sequenced was made available for each individual analysed.

Datasets found to be shared were further classified into four modalities according to the way in which data were found to be released:

- Body text—Data are provided in the main text of the article (e.g. tables, appendices or inferred from textual information)
- Online downloadable files—Data may be downloaded from institutional or personal sites.
- Supplementary material—Data are provided as supplementary tables, graphs or text available online in the journal’s or author’s web site
- Primary online databases—Data are available in widely disseminated and highly formalized technical infrastructures that enable their long term preservation and provide quality control procedures (i.e. GenBank, DDBJ and EMBL).

The actual availability of data as online material for modalities ii-iv was verified by visiting the relevant URLs (accessed on February 2013).

Differently from Milia et al. [36], when a dataset was shared in more than one modality (e.g. Online primary databases and supplementary material), only the most “effective” one was counted. Taking into account criteria of accessibility and preservation, depositing data in online primary databases was regarded as the best sharing modality, followed by supplementary material, online downloadable files and body text (S1 Table). When a dataset was composed of two different types of markers shared in different modalities (e.g. for mtDNA HVR1 sequences and coding region SNPs shared in online databases and body text, respectively), a value of 0.5 was assigned to each of them.

On the other hand, we identified two modalities of withholding datasets (i) complete data unavailable (applicable only for unilinear polymorphisms): both SNP and microsatellite (or SNP and sequencing) haplotypic data were available, but the information needed to reconstruct compound SNP/microsatellites (or SNP/sequencing) haplotypes was not given; (ii) only statistics-derived data available.

### Questionnaire-based survey

In order to gain further insights into the sharing behavior among researchers working with ancient human DNA, we asked first, last and corresponding authors of the papers inspected to answer some questions. Firstly, we collected information regarding their experience with ancient and modern DNA analysis. Secondly, we asked them to answer the following question: “Focusing on your overall publication experience, what is the contribution of the following factors to your choice of sharing ancient human DNA data?”. Respondents were given the possibility to rate the following statements in four ways (“not important at all”, “not very important”, “important” and “very important): (i) Compliance with policies of scientific Journals, funding bodies or other stakeholders; (ii) Expectation to receive a higher number of citations; (iii) Awareness of the importance of making my own study open to scientific inquiry and (iv) Awareness that data sharing should be common practice which all researchers ought to comply with to foster scientific progress. Finally, we asked researchers to answer the question “What is the contribution of the following factors to the higher rate of data sharing in DNA studies of ancient compared to extant humans?” giving marks to the following statements: (i) The need to comply with more stringent policies of funding bodies and/or journals; (ii) The greater need to make data and results open to scientific inquiry; (iii) Lack or lesser weight of ethical/privacy constraints.

The survey was carried out using Google forms (http://www.google.com/forms/about/). Responses received were completely anonymous since no personal information was asked.

## Results and Discussion

### Data sharing in human paleogenetics

We inspected a total of 207 datasets regarding mitochondrial, Y chromosomal and autosomal polymorphisms, reported in 162 papers (published from 1998 to 2013) which had been selected using a key-word-driven PubMed search. Mitochondrial datasets are the most numerous (132, 63.8% of the total), and encompass SNP, control region sequences and coding region/complete genomes. Y chromosomal datasets (28, 13.5% of the total) comprise SNP and microsatellite polymorphisms. Finally, autosomal datasets (47, 22.7%) include SNP, microsatellite and sequencing data, the latter being produced by next-generation sequencing technologies (S2 Table for more details). The datasets predominantly regarded *Homo sapiens* (172, 83.1%) compared to *Homo neanderthalensis* (32, 15.5%) and “*Homo denisovensis*” (3, 1.4%; S3 Table for further details).

**Fig 1:**
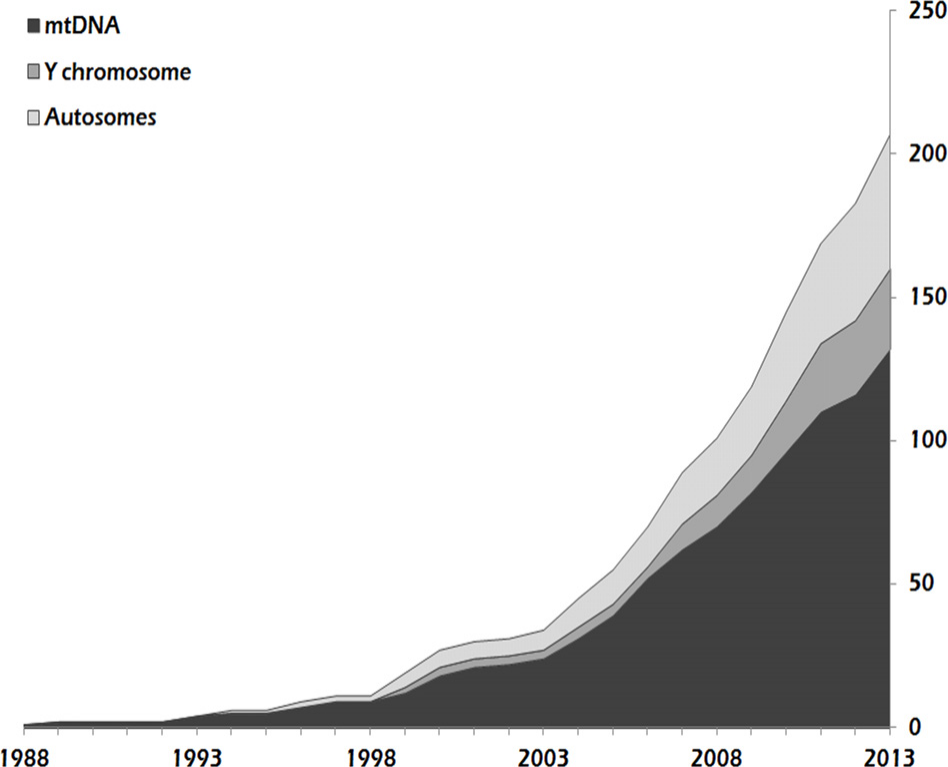
Cumulative distributions of papers on ancient human DNA from 1988 to 2013 according to the genetic system investigated.

The yearly distribution of published datasets shows that since 1988, mtDNA has been, and still is, the most frequently used genetic system (Fig. 1). The use of autosomal and Y-chromosomal loci started to increase from 2003 and 2006, respectively.

Two hundred and two datasets (97.6% ± 2.1%) were found to have made their genetic information fully available and reusable (Table 1), with little variation among genetic systems (96.4% ± 6.9% for Y chromosome; 97.7% ± 2.5% for mtDNA; 97.9% ± 4.1% for autosomes). Presenting only data-derived statistics was the main modality of withholding data. Interestingly, the five withheld datasets were published in the last six years: one dataset in 2008, two datasets in 2011 and two datasets in 2013.

**Table 1:** Data sharing modalities in human paleogenetics.

	mtDNA	Y chromosome	autosomes	Total
*Shared datasets*				
Online Primary databases	21.6% (27.5)	-	19.6% (9)	18.1% (36.5)
Supplementary material	21.6% (27.5)	29.6% (8)	27.1% (12.5)	23.8% (48)
Online downloadable files	-	-	2.2% (1)	0.5% (1)
Body text	57.4% (74)	70.4% (19)	51.1% (23.5)	57.7% (116.5)
*Withheld datasets*				
Complete individual data unavailable	33.3% (1)	-	-	20.0% (1)
Only data derived statistics available	66.7% (2)	100.0% (1)	100.0% (1)	80.0% (4)

Absolute counts are in parentheses.

In addition to the estimates of sharing rates, we investigated how data are made available. It should be noted that we chose to consider all main modalities of data sharing observed in our dataset (body text, online primary database, supplementary material, online downloadable files), rather than focusing on a specific one (e.g. see [37–39]). In all genetic systems, more than half of datasets are shared using body text, while supplementary material is used in a portion ranging from one fifth to one third of the total (see Table 1). About one fifth of mitochondrial and autosomal data is shared using online tools, mainly primary databases (e.g. GenBank) and, to a much lesser extent, downloadable files (see Table 1). However, both these modalities were not encountered for Y chromosome datasets. Although it is evident that the most frequently used sharing modalities do not ensure the highest degree of data findability, accessibility, useability and preservation (S1 Table), more positive signals can be observed when looking at their cumulative distributions from 1988 to 2013 (S1 Fig.). In fact, it is evident that the use of primary databases for mitochondrial and autosomal polymorphisms in human paleogenetics started to increase in 2006 and 2011, respectively—which coincides with the first application of complete mitochondrial and next-generation sequencing in human paleogenetics—and their use prevailed over other sharing modalities in 2013. This trend is expected to continue in the future due to the likely increase in the use of new sequencing technologies, whose larger amount of data necessarily requires digital archiving.

**Fig 2:**
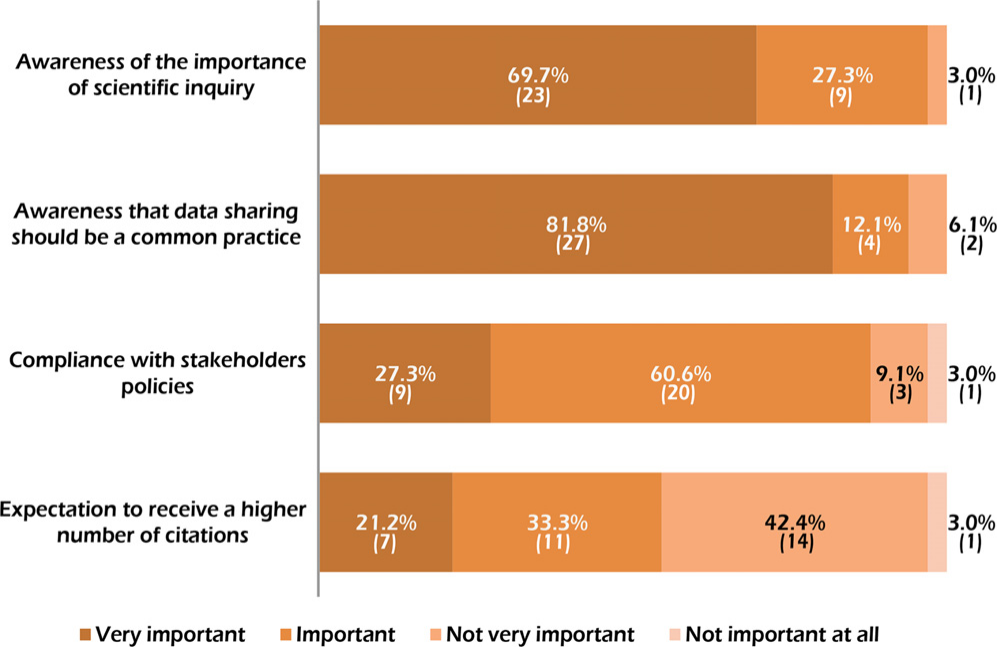
Results of the questionnaire-based survey. Rates of responses to the question “Focusing on your overall publication experience, what is the contribution of the following factors to your choice of sharing ancient human DNA data?”. The absolute values are given in parentheses. See Materials and Methods for complete statements.

As a complement to the analysis of data retrievability from published papers, we asked the authors of inspected papers to give a mark concerning four possible factors that influence their decision on whether to share data or not (Fig. 2). Although we received valid responses from only a part of the researchers emailed (33 respondents, corresponding to 24.0% of the total sample), the results seem worthy of discussion.

The vast majority of respondents indicated the importance of “making my own study open to scientific inquiry” (97.0% of respondents) and the awareness that “data sharing should be a common practice in scientific research” (93.9%) as the main reasons for making their data freely available to others. A slightly lower percentage (87.9%) pointed to the need to “comply with the sharing rule of Journals, funding bodies or other stakeholders” but only one third of them considers this as a very important factor which influenced their choice to share. Finally, the expectation to receive a higher number of citations seems to have played only a minor role. Even with the caution which is necessary due to the fact that the researchers’ ethos is called into question (see the “social desiderability bias” in Bowling 2005 [40]), these results suggest that the high sharing rate observed in human paleogenetics cannot be simply explained by the need to comply with norms or expectations of any scientific reward. This is also supported by the fact that a substantial part of papers (44.4%) was published in Journals in which data sharing is not mandatory. On the other hand, a look at the historical evolution of human paleogenetics supports the idea that epistemological motivations might have played a not negligible role in the observed sharing behaviour (see the “What human paleogenetics can teach the Open Science movement” section.)

### A comparison among different fields of genetic research

In order to better appreciate the meaning of the results obtained in the course of this study, data for human paleogenetics were compared with those of Milia et al. [36] for human evolutionary, forensic and medical genetics. This comparison is particularly appropriate for two reasons. First, the two studies were carried out using the same criteria for paper selection, definition of “data”, criteria to define shared and withheld datasets and following an identical workflow (see [36], pages 2–3). Second, the four research fields share not only most of their methodologies (based on DNA typing and sequencing), but also three important conditions which should favour data sharing: (i) the codified nature of genetic information; (ii) simplicity of basic metadata; (iii) availability of infrastructures for storage and dissemination. Thus, a number of confounding factors may be excluded.

**Fig 3:**
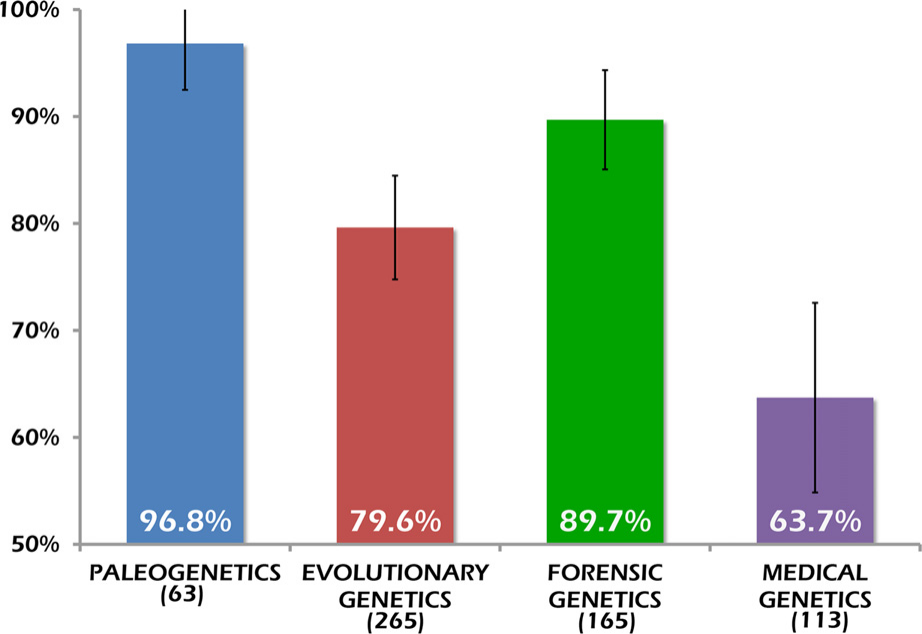
Sharing rates in papers concerning mitochondrial and Y chromosomal polymorphisms in humans. Vertical bars indicate 95% confidence intervals. The total number of scrutinized datasets for each field of research is reported in parentheses. All papers were indexed in Medline from 1/1/2008 to 31/12/2011.

As shown in Fig. 3, the sharing rate for human paleogenetics (recalculated to match exactly the genetic systems and period of data collection of Milia et al. [36]) is the highest (96.8%) and in two comparisons (with medical and evolutionary genetics) the difference is statistically significant (alpha = 0.05). Unfortunately, no comparison with other empirical studies is possible since the definition of data, inclusion criteria and workflow varied substantially among studies.

**Fig 4:**
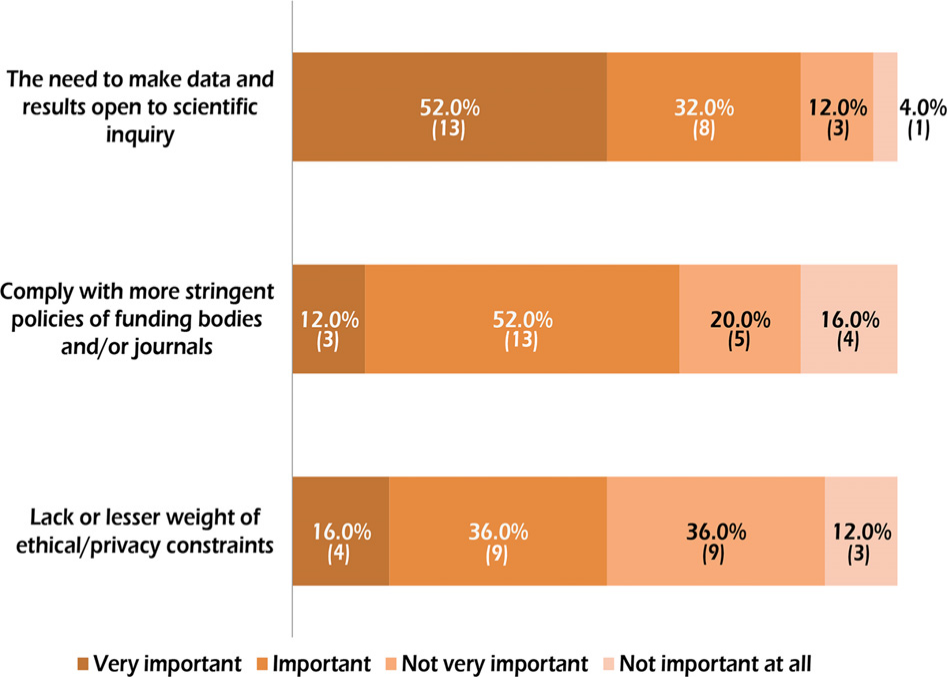
Results of the questionnaire-based survey. Rates of responses to the question “What is the contribution of the following factors to the higher rate of data sharing in DNA studies of ancient compared to extant humans?”. The absolute values are given in parentheses. See Materials and Methods for complete statements.

The results of the questionnaire-based survey turned out to be useful to gain insights into the difference observed in the sharing rate estimated in this study and in Milia et al. [36] (see Fig. 4). When we asked authors of surveyed papers that had also worked with extant populations (a total of 25 respondents) what reasons can explain the higher sharing rate of ancient DNA datasets, a large portion of respondents (84.0%) indicated “the greater need to make data and results open to scientific inquiry” as an important or very important factor. On the other hand, the answers “The need to comply with more stringent policies of funding bodies and/or journals” and “lack or lesser weight of ethical/privacy constraints”, received lesser consideration, with 64.0% and 52.0% of respondents marking them as important or very important. Once more, the strong awareness of the importance of scientific inquiry seems be a key factor for scholars working on ancient human DNA.

**Fig 5:**
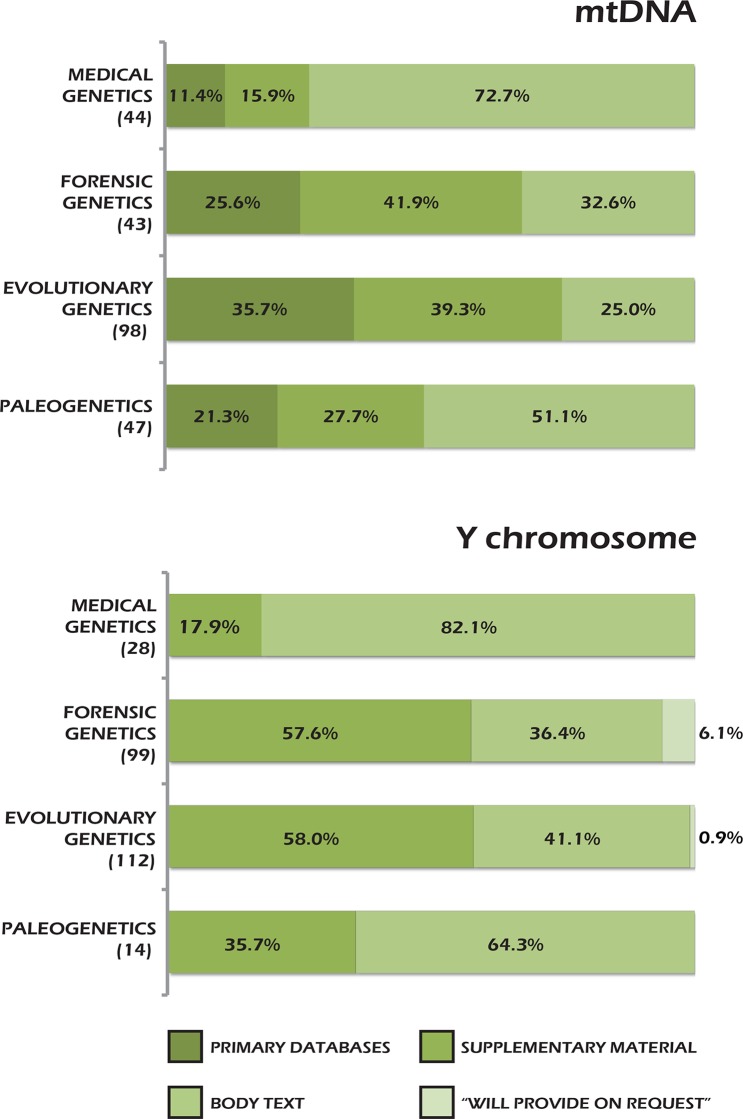
Frequencies of sharing modalities in the four genetic research fields analyzed. Rates of usage of different sharing modalities based on the inspection of papers indexed in Medline from 01/01/2008 to 31/12/2011. The total number of scrutinized datasets for each field of research is reported in parentheses. It should be noted that the modality “will provide on request” was observed only by Milia et al. [32].

Other useful insights are provided by the comparison of sharing modalities. As shown in Fig. 5, only in medical genetics did we observe a more frequent use of body text (for both mtDNA Y and chromosome data) and a less frequent use of primary databases than in human paleogenetics. On the other hand, evolutionary genetics appears to be the field where the adopted modalities (mostly primary databases and supplementary material) ensure the highest degree of findability, accessibility, useability and preservation despite its relatively low sharing rate. Thus, it appears that widespread data sharing does not necessarily coincide with a prevalent use of best sharing modalities, evidence which points to the need to look simultaneously at both aspects in future empirical studies.

Looking more closely at the features of the primary databases helps us understand what is probably the main reason for the gap between the modalities of sharing data which are actually practiced and the best available. We should consider, in fact, that the microsatellite and SNP polymorphism data we are dealing with were produced by using methods which evaluate fragment length or allelic status at specific nucleotide positions, respectively. Unfortunately, the resulting information cannot be deposited in primary databases since they are suitable only for sequence data or SNP data produced with microarray technologies. It follows that depositing in primary databases is possible only for mtDNA sequencing data (e.g. hypervariable region sequences, complete mitochondrial genomes), but unfeasible for the Y chromosomal data taken into consideration since they all refer to SNP and/or microsatellite polymorphisms. Therefore, implementing the submission of microsatellite and SNP data in GenBank and interoperating databases is worth taking into consideration as a means to increase data findability, accessibility, useability and preservation in all the fields of genetic research studied here.

### What human paleogenetics can teach the Open Science movement

We believe that our analysis of data sharing in human paleogenetics conveys three important messages to all those who are interested in increasing the openness of research data.

First, we provide evidence that awareness of the importance of transparent scientific practices may help achieve a very high data sharing rate. Certainly, policies and rules of funding bodies, academic institutions and scientific publishers may be very effective when dealing with specific projects or papers published in specific journals [7, 14, 15, 41]. However, our results suggest that epistemological motivations may effectively complement external policies when we move to a broader unit of observation, such as research fields where norms and incentives to share data are not necessarily always at work. This points to the need to make all players in scientific research conscious of the importance of open data to improve quality and reproducibility of research products [42]. We sustain that a key step to achieve this goal is in the education of young researchers regarding the principles of Open Science, so as to make them understand its connections with scientific progress and appreciate the importance of transparency and trust in research [19, 43–46]. Human paleogenetics may serve as an excellent case study for all these purposes.

Second, from what we observed for different fields of human genetic research, a very high sharing rate is not necessarily associated with the preferential use of archiving tools which make data more easily accessible, findable, useable and better preserved. Therefore, attention should be paid not only to the rate but also to the modality in which data are shared. We have shown that by taking into account all the different modalities of sharing data (body text, supplementary materials, online primary databases and online downloadable files), we may obtain a more complete assessment of the scientific practices and understand what the most important barriers are to a robust and effective data sharing. This latter point is well exemplified by the detrimental effect on the use of the best sharing modalities due to the unavailability of primary databases for specific types of polymorphisms.

Third and finally, the case of human paleogenetics provides an example of how data openness and transparency may play an important role in the development of specific research fields. The particular attitude of researchers working with ancient human DNA towards data sharing can probably be better understood by briefly looking at the history of their research field. Pioneered by Svante Pääbo [47] in mid 80’s, this field immediately attracted great interest due to its potential in shedding light on key issues of human evolution [48]. However, its development was hampered by controversies surrounding the time of DNA preservation and the risk of contamination during excavations and laboratory procedures [49, 50]. In fact, the DNA sequences obtained from a 2,400-yr-old mummy by Pääbo [47] using molecular cloning is today considered to be a result of contamination [51]. More in general, the field of human paleogenetics was considered by many to be untrustworthy until the application of next-generation sequencing [52]. Nonetheless, human paleogenetics is today a small but absolutely dynamic research field, which takes advantage of next-generation sequencing techniques to increase its analytical power. This includes testing for contamination, and attracts particular interest from the scientific community and the public [53, 54]. We argue that openness of researchers to the scientific scrutiny of their data coupled with the adoption of stringent standards and cross-laboratory validation procedures has been crucial in overcoming doubts concerning scientific rigor and data reliability [51]. In this way, human paleogenetics avoided the decline which occurred with other promising approaches adopted to study the remote human evolutionary past, such as DNA-DNA hybridization [55], where lack of reproducibility was a critical aspect. Thus, the case of human paleogenetics illustrates that data sharing and, more in general, openness to scientific inquiry, can help build rigorous and reliable scientific practices even in the presence of complex experimental challenges.

## Supporting Information

S1 Dataset Information collected on datasets analyzed in the course of this study. Na = information not available.
(XLSX)

S2 Dataset Answers to the questionnaire. (XLS)

S1 Fig Cumulative distributions of different sharing modalities from 1988 to 2013 according to the genetic system investigated. (TIF)

S1 Table Efficacy of different data sharing modalities in terms of findability, accessibility, useability and preservation. (XLSX)

S2 Table Characterization of the datasets under scrutiny in terms of genetic polymorphisms. (XLS)

S3 Table Characterization of the datasets under scrutiny in terms of species. (XLS)
