# Supplementary figures and images for: When Data Sharing Gets Close to 100%: What Human Paleogenetics Can Teach the Open Science Movement
Source: PLoS One. 2015 Mar 23;10(3):e0121409. doi: 10.1371/journal.pone.0121409 (PMC4370607; doi:10.1371/journal.pone.0121409)

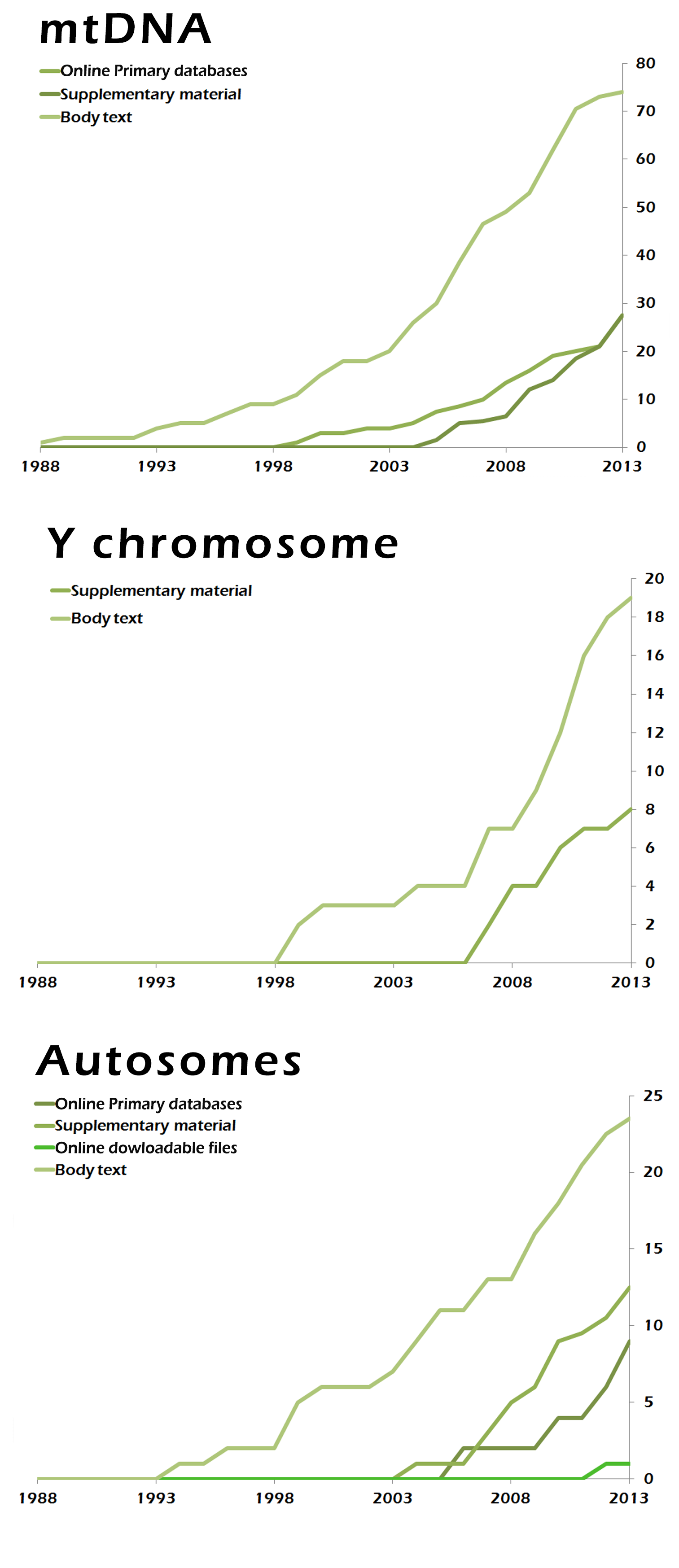

Supplement: S1 Fig — (TIF) [file pone.0121409.s003.tif]
